# Supplementary material for: Splice-Junction-Based Mapping of Alternative Isoforms in the Human Proteome
Source: Cell Rep. Author manuscript; Available in PMC 2020 Jan 15. (PMC6961840; doi:10.1016/j.celrep.2019.11.026)
Supplement: 3 [file NIHMS1546469-supplement-3.zip › DF2/PXD000561/AdrenalGland-25-Q96HR9-NVKPSQTPQPK.pdf]

A

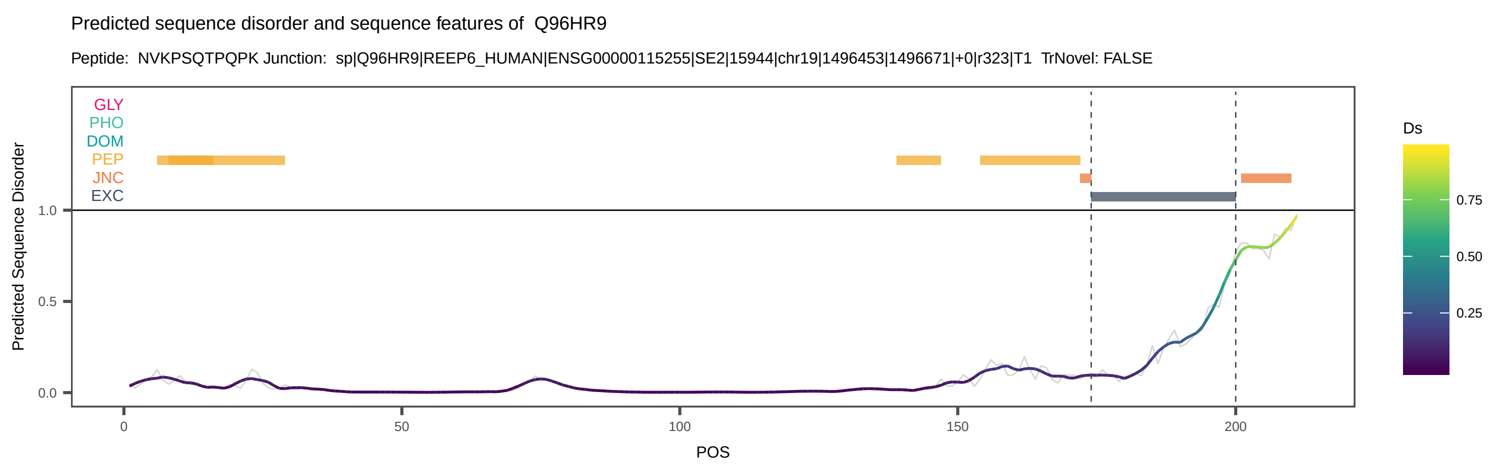
Predicted sequence disorder and sequence features of Q96HR9
Peptide: NVKPSQTPQPK Junction: sp|Q96HR9|REEP6_HUMAN|ENSG00000115255|SE2|15944|chr19|1496453|1496671|+0|r323|T1 TrNovel: FALSE
GLY
PHO
DOM
PEP
JNC
EXC
Predicted Sequence Disorder
1.0
0.5
0.0
0
50
100
150
200
POS
Ds
0.75
0.50
0.25

B

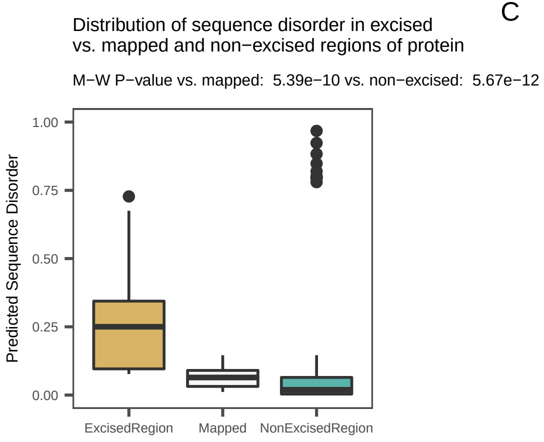
Distribution of sequence disorder in excised
vs. mapped and non−excised regions of protein
M−W P−value vs. mapped: 5.39e−10 vs. non−excised: 5.67e−12
Predicted Sequence Disorder
1.00
0.75
0.50
0.25
0.00
ExcisedRegion
Mapped
NonExcisedRegion

C
